# Immediate outcome prognostic value of plasma factors in patients with acute ischemic stroke after intravenous thrombolytic treatment

**DOI:** 10.1186/s12883-022-02898-6

**Published:** 2022-09-20

**Authors:** Huanhuan Lu, Siyi Li, Xin Zhong, Shuxuan Huang, Xue Jiao, Guoyong He, Bingjian Jiang, Yuping Liu, Zhili Gao, Jinhong Wei, Yushen Lin, Zhi Chen, Yanhua Li

**Affiliations:** 1 Department of Neurology, The People’s Hospital of Guangxi Zhuang Autonomous Region, Institute of Brain and Mental Diseases, Guangxi Academy of Medical Sciences, Nanning, China; 2 Youjiang Medical University For Nationalities, NO.98 ChengXiang Road, Baise, 533000 China

**Keywords:** Acute ischemic stroke, Intravenous thrombolysis, Plasma factors, NIHSS score, Prognosis

## Abstract

In the present study, we explored multiple plasma factors to predict the outcomes of patients with AIS after IVT. Fifty AIS patients who received IVT with alteplase were recruited and divided into two groups according to their NIHSS scores. Serum from all subjects was collected to quantitatively analyze the levels of different plasma factors, IL-6, MMP-9, ADAMTS13, TNC, GSN and TRX, using Luminex assays or ELISA measurements. Compared with the levels assessed at the onset of AIS, the levels of MMP-9 (*P* < 0.001), ADAMTS13 (*P* < 0.001), and TRX (*P* < 0.001) significantly decreased after IVT. The level of IL-6 was significantly increased in the NIHSS > 5 group at admission (*P* < 0.001) compared to the NIHSS ≤ 5 group. AIS patients with a poor prognosis had lower levels of ADAMTS13 at 72 h post-IVT compared with patients with a good prognosis (*P* = 0.021). IL-6 also was notably higher in the poor outcome group (*P* = 0.012). After adjusting for confounders, ADAMTS13 at 72 h post-IVT was an independent protective factor for prognosis in AIS patients with an adjusted OR of 0.07 (*P* = 0.049), whereas IL-6 was an independent predictor of risk for AIS patients with an adjusted OR of 1.152 (*P* = 0.028). IVT decreased MMP-9, ADAMTS13, and TRX levels in the plasma of AIS patients. Patients with a NIHSS score of less than 5 exhibited lower IL-6 levels, indicating that increased levels of IL-6 correlated with AIS severity after IVT. Therefore, IL-6 and ADAMTS13 might be useful plasma markers to predict the prognosis in AIS patients at 90-days after IVT.

## Introduction

Stroke is a leading cause of death and the primary cause of disability worldwide, with ischemic stroke accounting for approximately 80% of the cases [1]. Intravenous thrombolytic treatment (IVT) using alteplase within a narrow time window is the leading treatment for acute ischemic stroke (AIS) [2]. However, the benefits of intravenous thrombolysis for recanalization are limited. Ischemia and hypoxia in brain tissue prior to recanalization can result in increased release of pro-inflammatory cytokines, leading to pathological damage, such as blood–brain barrier disruption, brain edema, and cell death. Current studies have suggested that neuroinflammation mediates neuronal damage, increasing neurological deficits during the acute phase of cerebral ischemia [3, 4]. Numerous studies have reported that different inflammatory and cardiogenic biomarkers (e.g., IL-6 and MMP-9) are closely associated with ischemic stroke and its functional prognosis [5–7]. Therefore, identifying validated plasma markers to predict the outcome of AIS after IVT is essential to help adjust treatment strategies for patients with AIS.

To explore the potential plasma markers that could be used to predict the AIS outcome after IVT, we prospectively collected peripheral blood from AIS patients before and after IVT to investigate the following plasma biomarkers, interleukin-6 (IL-6), matrix metalloproteinase 9 (MMP-9), a disintegrin and metalloproteinase with a thrombospondin type 1 motif member 13 (ADAMTS13), tenascin-C(TNC), gelsolin(GSN), and thioredoxin (TRX). In addition, the relationship between dynamic changes before and after IVT, disease severity, and the predictive value of the plasma markers to assess functional outcomes also were analyzed.

## Materials and methods

### Patient selection

Patients with AIS admitted to the Department of Neurology at the People's Hospital of Guangxi Zhuang Autonomous Region for IVT between January 2020 and June 2021 were prospectively entered into the study. The study was performed according to the World Medical Association (WMA) Declaration of Helsinki and approved by the Ethics Committee of Guangxi Zhuang Autonomous Region People's Hospital. Given its observational and anonymous nature, the need for patient consent to participate in this study was waived.

The inclusion criteria were as follows: (1) AIS patients who underwent IVT; (2) age was ≥ 18 years; (3) the initial modified Rankin Score (mRS) on admissions ≤ 2.

The exclusion criteria were as follows: (1) unfavorable outcomes occurred before sampling; (2) the patient exhibited significant pre-stroke disability (pre-stroke mRS ≥ 2); (3) evidence of intracranial hemorrhage, subarachnoid hemorrhage, arteriovenous malformation, aneurysm, or intracranial tumor verified by CT/MRI on admission; (4) the presence of pre-existing neurological or psychiatric disease that would confound the neurological evaluation; (5) active or recent hemorrhage and a history of trauma or surgery within two months before stroke onset; (6) concurrent infection at the time of sample collection; (7) the presence of rheumatoid immune diseases, severe liver or kidney disease, hematopathy, or malignant tumors; (8) suspicion of the presence of an infectious embolus or bacterial endocarditis; (9) a baseline platelet count < 50,000/μL; (10) the patient was pregnant or lactating; (11) missing clinical, imaging, or follow-up data or information; and (12) blood samples were of poor quality.

Ninety-five consecutive patients were included in the study. Of the 95 patients, 15 patients were excluded due to the presence of infection on admission, 2 patients had malignant tumors, 1 patient had a rheumatic immune disease,2 patients had incomplete clinical data and missing follow-up data, and 1 patients’ blood samples were of low quality. The remaining eligible patients (*n* = 74) were divided into two groups based on their NIHSS scores. One group consisted of patients with a NIHSS score greater than 5 (NIHSS > 5) (*n* = 36), and the other group of patients had a NIHSS score equal to or less than 5 (NIHSS ≤ 5) (*n* = 38).

### Clinical information

IVT was carried out according to international guidelines. 0.9 mg alteplase/kg body weight was administered with a maximum dose of 90 mg. The drug was administered as an infusion of a 10% bolus dose within 1 min, followed by an infusion of a 90% dose within 60 min. The NIHSS score was assessed at admission and at 24 h and one week after stroke onset to determine neurological function. Baseline demographic information and vascular risk factors were collected from the patients, including baseline stroke severity (NIHSS score at admission), pre-stroke mRS, cerebrovascular risk factors (age, sex, hypertension, diabetes, hyperlipidemia, atrial fibrillation, coronary artery disease, atherosclerosis, as well as smoking and alcohol consumption status). Systolic blood pressure (SBP) and diastolic blood pressure (DBP) were measured three times within 24 h of admission, and the mean systolic and mean diastolic blood pressures were calculated. Biochemical parameters, including liver function, coagulation, uric acid, creatinine, and other laboratory tests, including blood cell count, were obtained before or 24 h after thrombolytic therapy. Computed tomography, magnetic resonance and digital subtraction angiography, electrocardiography, and carotid ultrasound were used to determine the stroke etiology. The clinical outcome at 90 days after AIS was assessed using the mRS. An mRS ≥ 2 or death was defined as a poor prognosis [8, 9]. The data were obtained using a telephone follow-up interview by a physician who was unaware of the patients’ clinical information and factor levels.

### Biomarker assay assessment

Blood samples were collected before stroke onset (t0), 24 h after thrombolytic therapy (t1), and 72 h after thrombolytic therapy (t2). The samples were immediately centrifuged at 3,000 g for 15 min (Thermo Scientific Haraeus Multifuge 3SR plus centrifuge, US) and stored at -80 °C until analyzed.

The levels of IL-6, MMP-9, TNC, and ADAMTS13 were determined using a Luminex R&D System kit (Labex Bio, Shanghai, China), following the manufacturer’s instructions. This method utilized different antibody factors covalently cross-linked to specifically coded microspheres. Each coded microsphere was dyed with a different fluorochrome in different ratios, resulting in a corresponding fluorescent code and assay. The coefficients of variation (CVs) were 0.56% for IL-6, 0.58% for MMP-9, 0.44% for TNC, and 0.3% for ADAMTS13.

The TRX and GSN antibodies in serum were determined using a quantitative sandwich enzyme immunoassay (ELISA), following the manufacturer's instructions (Service Bio, Wuhan, China). The CVs for the TRX inter-assay and intra-assay ranged from 8 to 10%, respectively. The lower detection limit was 1.172 ng/mL, and the line range was 4.688 ng/ml to 300 ng/ml. The CVs for the GSN inter-assay and intra-assay ranged from 8 to 10%, respectively, and exhibited a range of 3.12 ng/ml to 200 ng/ml with a lower detection limit of 0.78 ng/ml.

### Statistical analysis

Statistical analysis of the data was performed using SPSS version 24.0 and Graphpad prism 8.0.2. Continuous variables that conformed to a normal distribution were expressed as means ± standard deviation. Other continuous variables that did not exhibit a normal distribution were expressed as medians and interquartile ranges (25% to 75%). Categorical variables were expressed as composition ratios. The plasma markers were labeled as continuous variables, log-transformed, and tested using repeated-measures ANOVA. The Pearson's chi-square test was used for categorical variables to test for differences in baseline characteristics. We also used binary logistic regression analysis to detect risk factors affecting the severity of illness and adverse prognosis. After correcting for potential confounders, including age and gender, independent influences associated with the prognosis of patients who underwent intravenous thrombolysis for AIS were screened using univariate and multivariate logistic regression analyses. Odds ratios (ORs) and 95% confidence intervals (CIs) were calculated for each endpoint. Subject (receiver) operating characteristic (ROC) curves were used to assess the plasma markers (IL-6, MMP-9, ADAMTS13, TNC, GSN, and TRX) and determine the optimal cut-off point, sensitivity, and calculated specificity.

## Results

### Clinical characteristics

Ninety-five patients were recruited, including 43 men and 52 women. Only 74 patients (43 males and 31 females) with adequate admission data were included in the study. The patients had a median NIHSS score on admission of 5.5(IQR 4 to 13). Patients were categorized into two subgroups according to their NIHSS scores. One group included patients with a NIHSS score high than 5 (NIHSS > 5; IQR 7.5 to 17.5, median: 13; *n* = 36). The other group included patients with a NIHSS score equal to or less than 5 (NIHSS ≤ 5; IQR 3 to 5, median: 4; *n* = 38) (Table 1).

**Table 1:** Clinical characteristics

Clinical characteristics	Total	NIHSS on admission		*p*-value
		NIHSS ≤ 5	NIHSS > 5	
Age, mean (SD),years	68.12 ± 9.215	71.08 ± 8.381	65.16 ± 9.214	0.022
Male, n	43	21	22	0.684
NIHSS 24 h,IQR	4(2–8.5)	3(2–4.5)	5(3–17)	0.006
Mean SBP, mean(SD),mmHg	140.5 ± 14.71	142.48 ± 13.198	138.52 ± 16.107	0.346
Mean DBP, mean(SD),mmHg	79.5 ± 11.53	77.92 ± 11.849	81.08 ± 11.217	0.338
Max SBP, mean(SD),mmHg	156.58 ± 25.237	158.04 ± 19.817	155.12 ± 30.053	0.687
Max DBP, IQR	89.5(79–102.25)	86 (77.5–98.5)	96 (79–108.5)	0.225
Hypertension, n	41	19	22	0.269
Diabetes mellitus, n	17	9	8	0.765
Atherosclerosis, n	47	24	23	0.552
Atrial fibrillation, n	5	1	4	0.157
Smoke, n	16	7	9	0.544
Alcohol, n	9	6	3	0.269
Congestive heart failure, n	3(	1	2	0.552
Coronary artery disease, n	11	4	7	0.306
Hyperlipidemia, n	21	8	13	0.152
mRS 90d, IQR	2.5(2–4)	2(1–3)	3(2–4)	0.008

Data are expressed numerically (as a percentage), as the mean ± standard deviation or as the median (minimum; maximum), as appropriate. *IQR* Interquartile range, *NIHSS* National Institute of Health Stroke Scale, *SBP* Systolic blood pressure, *DBP* Diastolic blood pressure

The mean age of the low NIHSS score group was significantly higher than that of the high NIHSS score group (*P* = 0.022). The median NIHSS score 24 h after admission was 4 (IQR 2 to 8.5). Patients in the high NIHSS score group (IQR 3 to 17, median: 5) had a significantly higher NIHSS score 24 h after admission than those in the low NIHSS score group (IQR 2 to 4.5, median: 3) (*P* = 0.006). The median mRS at 90 days was 2.5(IQR 2 to 4), and the mRS at 90 days was significantly higher in the high NIHSS score group (IQR 2 to 4, median: 3) than in the low NIHSS score group (IQR 1 to 3, median: 2) (*P* = 0.008). There were no significant differences in hypertension, diabetes, hyperlipidemia, atrial fibrillation, coronary heart disease, arteriosclerosis, mean systolic and diastolic blood pressure, smoking, and drinking between the two groups.

#### Changes in plasma factors before and after IVT in patients with AIS

**Fig. 1:**
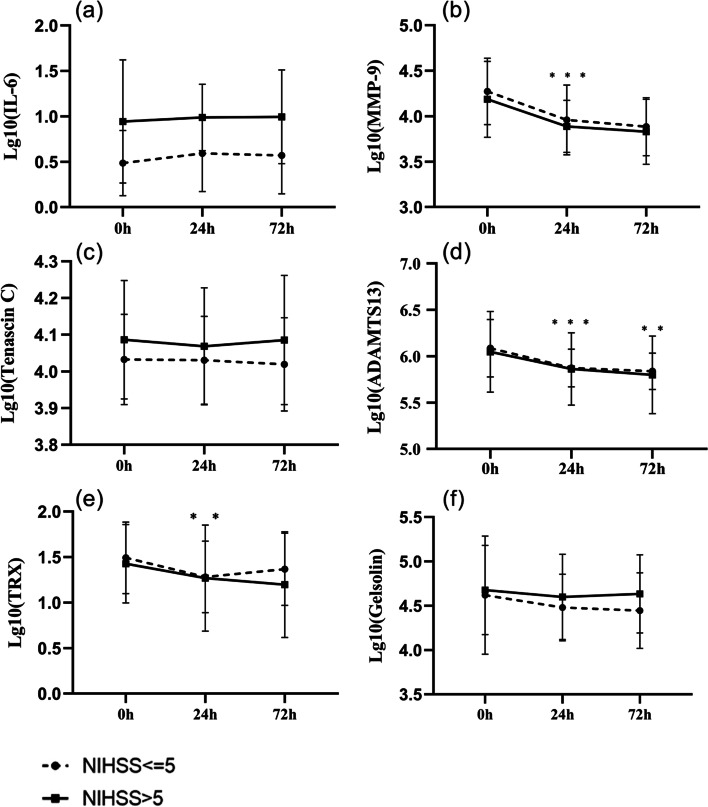
Changes and intergroup differences in IL-6 (**a**), MMP-9 (**b**), TNC (**c**), ADAMTS13 (**d**), TRX (**e**), GSN(f) at different time points after acute ischemic stroke in patients admitted with mild disease and those admitted with reorganized disease.* *p ≤ 0.01,***p ≤ 0.001)

MMP-9, ADAMTS13, and TRX levels were correlated with significant decreased in pre-IVT patients compared to 24 h after IVT (*P* < 0.05). The level of ADAMTS13 remained significantly decreased at 72 h post-IVT compared to 24 h after IVT (*P* = 0.008). The levels of IL-6, TNC, and GSN were not significantly different among the patients when assessed pre-IVT or at 24 h and 72 h after IVT (*P* > 0.05) (Fig. 1 and Table 2).

**Table 2:** Changes in plasma markers before and after treatment with IVT in patients admitted with reconstituted disease and admitted with mild disease group

		lg10(IL-6)	lg10(MMP-9)	lg10 (TNC)	lg10 (ADAMTS13)	lg10 (TRX)	lg10 (GSN)
Overall analysis	HF	0.684	0.99	1	0.696	0.944	0.661
NIHSS ≤ 5 vs NIHSS > 5	F	15.401	1.105	1.76	0.107	0.575	0.993
	P	0.000^a^	0.298	0.191	0.745	0.452	0.324
t0 vs t1	F	1.196	18.505	1.35	26.694	9.441	3.047
	P	0.28	0.000^a^	0.25	0.000^a^	0.003^a^	0.087
t1 vs t2	F	0.038	1.445	0.087	7.55	0.008	0.000
	P	0.847	0.235	0.769	0.008^a^	0.929	0.992

*HF* Huynh–Feldt Epsilon, *NIHSS* National Institute of Health Stroke Scale, *NIHSS* ≤ *5 NIHSS* on admission ≤ 5; NIHSS > 5, NIHSS on admission > 5

^a^The overall analysis was a two-factor repeated measures ANOVA

#### Relationship between plasma factors and NIHSS scores at admission

A NIHSS score greater than 5 at admission is thought to indicate the presence of severe neurological deficits. Therefore, the relationship between stroke severity and plasma factors was analyzed further. Univariate analysis demonstrated that the IL-6 level was significantly increased at admission in the NIHSS > 5 group compared to the NIHSS ≤ 5 group (*P* < 0.001), indicating a positive correlation between the level of IL-6 and the severity of the illness at admission. However, there were no significant differences in the levels of MMP-9, ADAMTS13, TRX, TNC, and GSN between the NIHSS > 5 and NIHSS ≤ 5 groups (Table 2).

#### Independent risk predictors of disease severity

After adjusting for age and gender, the multifactorial logistic analysis revealed that the level of IL-6 before IVT still was significantly associated with the NIHSS score at admission (OR 219.963, 95% CI 8.039–6018.562, *P* = 0.001) (Table 3), suggesting that the pre-IVT IL-6 level was an independent predictor of disease severity in patients with AIS. In addition, the pre-IVT MMP-9 level (OR 0.046, 95% CI 0.005–0.958, *P* = 0.046) also correlated with the NIHSS score on admission after adjustment.

**Table 3:** Multifactorial association of plasma markers with disease severity at different time points after correction

Variables	B	S.E	Wald	*p*	OR	95%CI
IL-6 (t0)	5.393	1.688	10.205	0.001	219.963	8.039–6018.562
IL-6 (t1)	4.884	1.601	9.307	0.002	132.129	5.732–3045.502
IL-6 (t2)	4.569	1.65	7.665	0.006	96.459	3.798–2449.899
MMP-9(t0)	-2.636	1.323	3.971	0.046	0.072	0.005–0.958
MMP-9(t1)	-0.38	1.455	0.068	0.794	0.684	0.04–11.841
MMP-9(t2)	-0.674	1.626	0.172	0.679	0.51	0.021–12.335
TNC (t0)	5.897	4.108	2.061	0.151	364.101	0.116–1,142,529.513
TNC (t1)	-2.412	3.677	0.43	0.512	0.09	0.00–120.76
TNC (t2)	10.334	4.286	0.664	0.415	30,774.241	6.924–136,787,756.4
ADAMTS13(t0)	-0.343	1.299	0.07	0.792	0.71	0.056–9.060
ADAMTS13(t1)	-0.055	1.549	0.001	0.972	0.947	0.045–19.709
ADAMTS13(t2)	-1.11	1.363	0.664	0.415	0.329	0.023–4.76
TRX(t0)	-1.8	1.181	2.323	0.127	0.165	0.016–1.673
TRX((t1)	0.671	0.919	0.534	0.465	1.957	0.323–11.844
TRX(t2)	-3.076	1.538	3.999	0.046	0.046	0.002–0.941
GSN(t0)	-0.036	0.735	0.002	0.96	0.964	0.228–4.071
GSN(t1)	2.071	1.268	2.668	0.102	7.93	0.661–95.134
GSN(t2)	2.359	1.456	2.625	0.105	10.582	0.61–183.696

#### The predictive significance of plasma markers for neurological function at 90 days after IVT

**Fig. 2:**
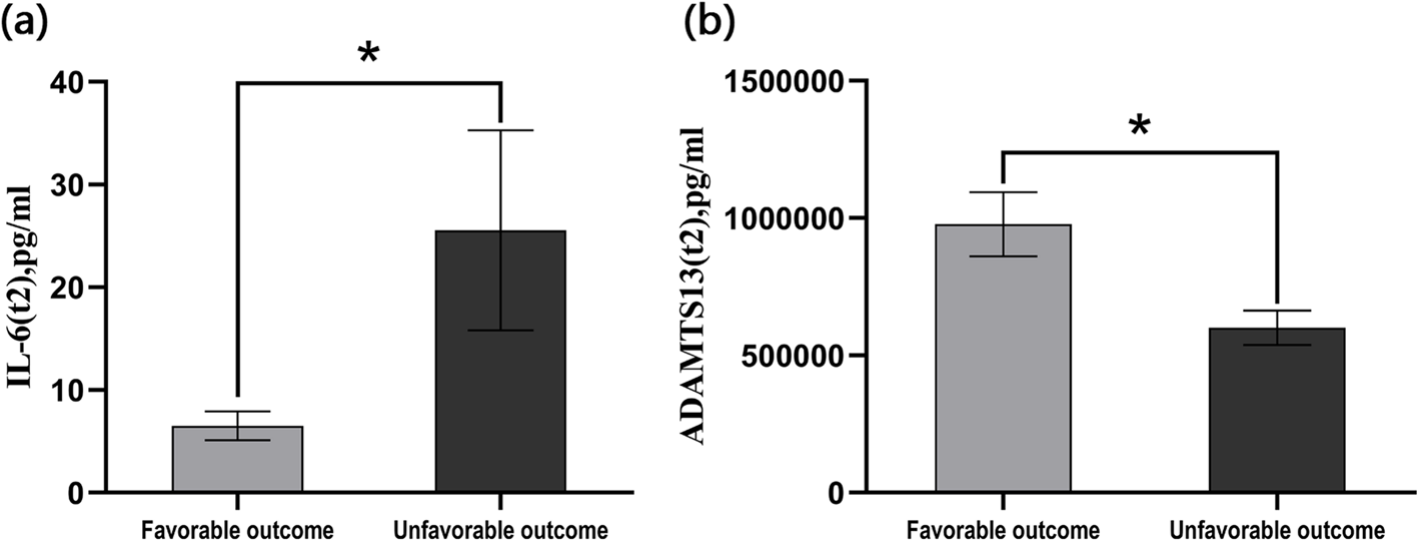
The relationship between IL-6, ADAMTS13 and AIS outcomes 72 h after IVT. The concentration of ADAMTS13 were decreased in the unfavorable outcome group. However, the levels of IL-6 were significantly increased in the poor outcome group compared with the favorable outcome group. Data are presented as mean ± SEM, *p < 0.05

At the 90-day follow-up examination, 21(28%) patients with AIS and IVT exhibited a poor prognosis. We found that serum ADAMTS13 levels at 72 h after IVT were significantly lower in AIS patients with poor outcomes compared with those with a good prognosis 551,181.00(IQR 440,539.00 to 788,283.50) pg/ml vs. 769,107.00(IQR 565,397.50 to 1,366,444.50) pg/ml, *P *= 0.021). The level of serum IL-6 at 72 h post-IVT was significantly higher in the poor outcome group [8.72 (IQR 3.04–26.89) pg/ml vs. 3.72 (IQR, 1.88–8.58) pg/ml, *P* = 0.012] (Fig. 2). However, the levels of MMP-9, TNC, GSN, and TRX at 72 h post-IVT were not significantly different between patients with a good prognosis and those with a poor outcome.

The prognosis of patients with AIS at 90-days after IVT was used as the dependent variable, and age, smoking, hypertension, diabetes, coronary artery disease, hyperlipidemia, atrial fibrillation, IL-6, MMP-9, ADAMTS13, TRX, TNC, and GSN were considered independent variables. Using univariate logistic regression analysis, we observed that the serum concentrations of IL-6 and ADAMTS13 at 72 h post-IVT were significantly associated with the functional outcome of AIS patients (Table 4). In multivariate logistic regression analysis after adjustment for other indicators, the serum concentration of ADAMTS13 at 72 h post-IVT functioned as a protective factor and was an independent predictor for functional outcome of AIS patients with an adjusted OR of 0.07 (95% CI, 0.005–0.991, *P* = 0.049), whereas IL-6 at 72 h post-IVT was an independent risk factor in functional outcome with an adjusted OR of 1.152 (95% CI,1.015–1.308, *P *= 0.028).

**Table 4:** Univariate and multifactorial associations of plasma markers with clinical prognosis

	Unadjusted OR(95%CI)	*p*	Adjusted OR (95% CI)	*p*
IL-6 (t2)	1.06 (0.996–1.127)	0.065	1.152 (1.015–1.308)	0.028
MMP-9 (t2)	0.818 (0.151–4.449)	0.816	-	-
ADAMTS13(t2)	0.093 (0.009–0.95)	0.045	0.07 (0.005–0.991)	0.049
TNC (t2)	4.711 (0.113–196.3)	0.415	-	-
TRX(t2)	2.072 (0.6–7.153)	0.249	-	-
GSN(t2)	2.545 (0.659–9.829)	0.175	-	-

*CI* Confidence interval, *OR* Odds ratio

**Fig. 3:**
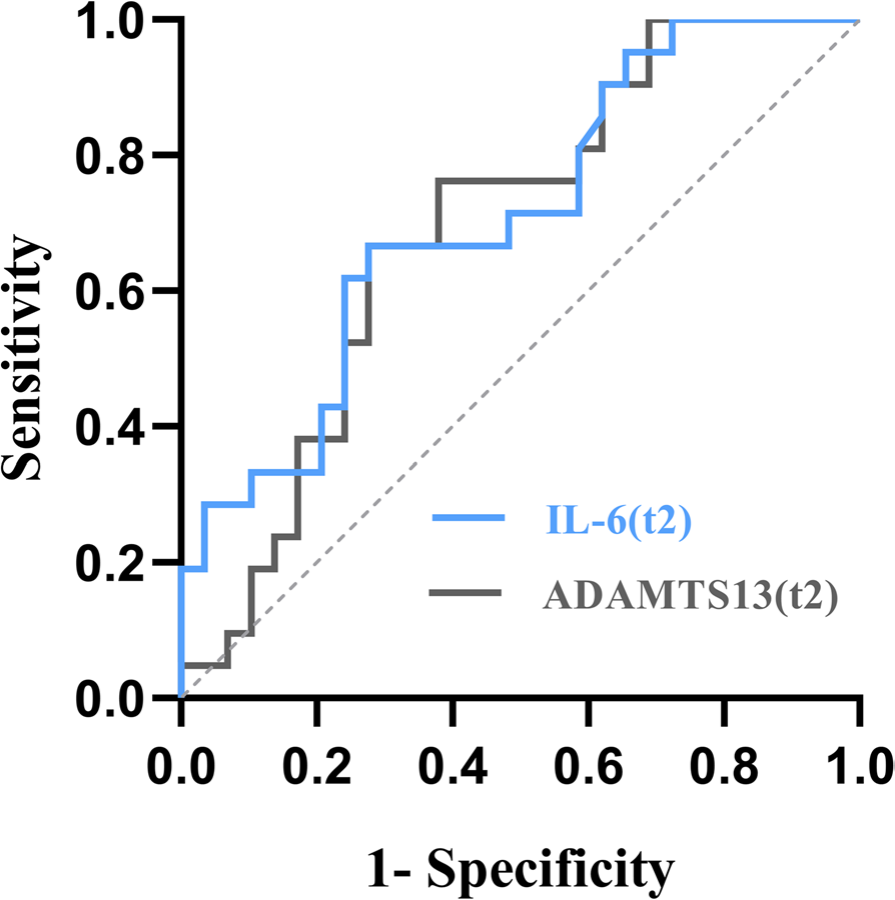
ROC curve of 90-day prognosis predicted by IL-6 and ADAMTS13 at 72 h after acute ischemic stroke onset. The optimal cutoff value of IL-6 was 5.795 pg/ml with a sensitivity of 66.7% and a specificity of 72.4% (AUC: 0.71, 95% CI (0.566–0.854; *P* = 0.012)

To assess the predictive effect of plasma markers on prognosis, we plotted ROC curves. The ROC curves for IL-6 and ADAMTS13 at 72 h post-IVT are shown in Fig. 3. ROC curve analysis determined that the best threshold value of IL-6 at 72 h post-IVT to predict the 90-day prognosis of AIS patients was 5.795 pg/ml, with a sensitivity of 66.7%, a specificity of 72.4%, and a Jorden's index of 0. 391; the area under the curve (AUC) was 0.71 (95% CI, 0.566 to 0.854, *P* = 0.012). The optimal cut-off value for the ADAMTS13 level at 72 h post-IVT was 634,010.5 pg/ml, with a sensitivity of 72.4%, a specificity of 66.7%, and a Jorden's index of 0.391; the AUC was 0.693 (95% CI, 0.547 to 0.839, *P* = 0.021), suggesting the level of IL-6 (AUC = 0.71, *P* = 0.012) exhibited a better predictive effect of a poor prognosis than the level of ADAMTS13 (AUC = 0.693, *P* = 0.021). Other plasma factors, including MMP-9 (AUC = 0.532, sensitivity 65.5%, specificity 57.1%, *P* = 0.702), TRX (AUC = 0.573, sensitivity 64.9%, specificity 88.3%, *P* = 0.382), TNC (AUC = 0.553, sensitivity 75.5%, specificity 67%, *P* = 0.523), and GSN (AUC = 0.608, sensitivity 78.7%, specificity 64.1%, *P* = 0.198) showed no significant differences between the mRS < 2 and mRS ≥ 2 groups (*P* > 0.05) (Fig. 3 and Table 5).

**Table 5:** ROC curve analysis of plasma biomarkers to predict poor prognosis

	AUC	Youden Index	Sensitivity,%	Specificity,%	*P*-value
IL-6 (t2)	0.71	0.391	66.7	72.4	0.012
MMP-9 (t2)	0.532	0.226	65.5	57.1	0.702
TNC (t2)	0.553	0.204	75.5	67	0.523
ADAMTS13 (t2)	0.693	0.391	72.4	66.7	0.021
TRX (t2)	0.573	0.193	64.9	88.3	0.382
GSN (t2)	0.608	0.297	78.7	64.1	0.198

*ROC* Receiver operating characteristic, *AUC* Area under the curve, *CI* Confidence interval

## Discussion

In our study, we evaluated the prognostic values of plasma factors in AIS patients who received IVT. We found that the levels of MMP-9, ADAMTS13, and TRX were significantly decreased in AIS patients with IVT. However, the levels of IL-6, TNC, and GSN showed no significant differences after IVT. When the AIS patients were divided into two groups based on their NIHSS scores, the IL-6 levels were significantly higher in the group with high NIHSS scores (NIHSS > 5). Moreover, the ROC curves revealed that IL-6 and ADAMTS13 levels could significantly predict the 90-day prognosis of AIS patients after IVT. These results indicated that IL-6 and ADAMTS13 might be independent factors that are able to predict patient prognosis at 90 days following IVT for AIS.

IL-6 is a critical cytokine involved in the acute phase of the inflammatory response. Numerous studies have shown that the level of IL-6 increases in cerebrospinal fluid (CSF) and peripheral blood after ischemic stroke [10–13]. In human studies, the serum level of IL-6 is significantly correlated with infarct size and survival in stroke [14, 15]. Francisco et al. [16] demonstrated that IL-6 emerged as the only biomarker independently associated with infarct volume in AIS patients. Similarly, in our study, the levels of IL-6 were increased in AIS patients with NIHSS scores greater than 5. Therefore, the IL-6 levels could be considered an independent factor in predicting the patients’ prognosis 90 days after IVT. Similarly, recent studies have confirmed that a high level of IL-6 after AIS was an independent predictor of poor functional outcomes [17–19]. Therefore, high IL-6 levels might partially contribute to poor functional outcomes and death [20].

Low levels of MMP-9 expression are expected in the normal brain [21]. However, the levels of MMP-9 are elevated after cerebral ischemia, and MMP-9 levels are closely related to the occurrence and development of cerebral infarction [21, 22]. High levels of MMP-9 can be detected in necrotic brain tissue and the ischemic penumbra following a stroke [23]. In the present study, the level of MMP-9 after IVT in AIS patients was lower than before IVT, which might indicate that the necrotic tissue decreased MMP-9 release due to the recanalization of blood vessels after IVT. The NIHSS scores were not correlated with the levels of MMP-9, suggesting that MMP-9 might not reflect disease severity or the amount of necrotic brain tissue present.

Similar to the changes in the levels of MMP-9, the levels of ADAMTS13 and TRX after IVT treatment in AIS patients were lower than before IVT. However, our results contradict the report of Xu et al. that showed that the level of ADAMTS13 was associated with the effects of endovascular treatment or thrombolysis after cerebral infarction in patients with acute stroke. In addition, high levels of ADAMTS13 were associated with arterial recanalization in that study [24]. Decreased levels of ADAMTS13 were associated with poor functional outcomes for AIS patients in this study [24, 25]. Specifically, our results demonstrated that the levels of ADAMTS13 at 72 h post-IVT functioned as a protective factor and was an independent predictor of functional outcome in AIS patients.

TRX is a ubiquitous protein with disulfide reductase activity and plays a critical role in cellular redox control and oxidative stress responses. Increased expression of TRX and TRXR significantly disturbs redox homeostasis [26]. In this study, we investigated for the first time changes in TRX levels before and after IVT and discovered that the level of TRX decreased in AIS patients after IVT. Therefore, we speculated that the reduced TRX levels indicated that the redox homeostasis might not be disturbed following IVT.

There were no significant differences in the levels of TNC and GSN between the NIHSS > 5 and NIHSS ≤ 5 groups before or after IVT. Bharath et al. [27] proved that TNC could induce post-stroke brain damage. The reason why we did not observe significant differences in TNC levels might be due to an insufficient time interval for blood collection. GSN is an actin-severing and capping protein that regulates actin assembly and might be involved in fibroblast activation. We did not observe significant changes in GSN before and after IVT, which might indicate that GSN is not involved in the occurrence and development of cerebral infarction.

Several limitations were associated with this study. The sample size was small, and the patients were recruited from a single center, thus the follow-up prognostic value is very strict. The IL-6, MMP-9, ADAMTS13, TNC, TRX, and GSN levels were detected at only two-time points. The follow-up and chronic-phase data were not available. ADAMTS13 activity and vWF antigen/activity has effects on thrombosis. However, in this study, we did not explore the relationship between ADAMTS13 activity and IVT patients’ outcomes. Finally, the NIHSS scores of patients with mild stroke might be subjectively influenced by physician. Thus, additional studies are necessary to address these issues.

## Conclusion

We found that the levels of MMP-9, ADAMTS13, and TRX were significantly decreased in AIS patients who received IVT. Patients with a NIHSS score greater than 5 exhibited higher levels of IL-6. The levels of IL-6 and ADAMTS13 significantly predicted the 90-day prognosis of AIS patients after IVT. Finally, the higher the IL-6 level, the worse the 90-day prognosis of patients who received IVT.

## Data Availability

All data generated or analysed during this study are included in this published article [and its supplementary information files.
